# Effect of chlorpyrifos on soil microbial diversity and its biotransformation by *Streptomyces* sp. HP-11

**DOI:** 10.1007/s13205-016-0462-2

**Published:** 2016-06-24

**Authors:** M. Supreeth, M. A. Chandrashekar, N. Sachin, N. S. Raju

**Affiliations:** Department of Studies in Environmental Science, University of Mysore, Manasagangothri, Mysuru, 570006 Karnataka India

**Keywords:** Chlorpyrifos, TCP, Diethylphosphorothioate, *Streptomyces sp.* HP-11, LC–MS

## Abstract

The application of pesticides in agricultural fields not only reaches the target pests but also with soil where it interacts with soil microorganisms resulting in change of microbial diversity. Chlorpyrifos (CP) is one such organophosphorous insecticide most widely used against various insects, termites, and beetles throughout the globe. In the present work, the effect of CP on soil microbial population was assessed by the cultivable method. The fertile soil which does not have a history of any pesticide application was treated with 100 and 200 µg/g of CP along with control which received only sterile water and incubated for 1, 7, and 14 days. The soil amended with the insecticide showed decrease in the number of colony forming units (CFU) of bacteria and fungi. However, *Streptomyces sp.* HP-11 which tolerated high concentration and also inhibited fungal population was further selected for biodegradation studies. After 14 days of incubation in Mineral salt media (MSM), the strain HP-11 biotransformed CP into 3, 5, 6-trichloro-2-pyridinol (TCP) and Diethyl Phosphorothioate (DETP), and its formation was confirmed by the *m*/*z* peak of LC–MS analysis, which was later metabolized to unknown polar metabolites. The results obtained highlights that the application of chlorpyrifos favored the *Actinomycete* growth in the soil, thereby inhibiting other microorganisms and the strain HP-11 harbors metabolic pathway for detoxification of CP and its hydrolysis product TCP into polar metabolites, thus suggesting the strain HP-11 will be a potential bioaugmenting agent for the bioremediation of chlorpyrifos contaminated soil and water.

**Electronic supplementary material:**

The online version of this article (doi:10.1007/s13205-016-0462-2) contains supplementary material, which is available to authorized users.

## Introduction

Pesticides are the synthetic compounds used to protect agricultural crops from disease causing pests. The applied pesticide will reach target pests by only 1 % and the remaining will come into contact with soil, where they undergo a variety of transformations that provide a complex pattern of metabolites (Andreu and Pico 2004). Fertility of soil is dependent on the soil microbial richness and diversity. As a natural decomposers, microorganisms enrich the soil nutrients, and improve soil texture and water holding capacity. Researchers have shown that the pesticides are always having their effect on the soil microorganisms. Some pesticides stimulate the growth of soil microorganisms and some have depressive effects or no effects. However, the relationship of different structures of pesticides on the growth of soil microorganisms is not easily predictable (Lo 2010).

Organophosphorous (OPs) insecticides are ester or thiol derivatives of phosphoric acid, whose mode of action is through the inhibition of enzyme Acetylcholinesterase, which is responsible for nerve transmission. Chlorpyrifos [*O*,*O*-diethyl *O*-(3,5,6-trichloro-2-pyridyl phosphorothiote)] is an important OPs insecticide, widely used against a broad spectrum of agricultural crops throughout the world at concentration of 3 to 15 kg/ha (Singh and Walker 2006). The extensive usage of chlorpyrifos having a half life from 10 to 120 days in soil has resulted in widespread environmental contamination affecting beneficial non-target soil microorganisms (Li et al. 2008).

Biotransformation is a process of modifying or cleavage of chemical structure of a parental molecule of an organism resulting in various metabolites depending on mechanism adopted by specific organisms. Biological systems, such as microorganisms, have been used to biotransform or detoxify pesticides. There are many reports on the biotransformation of chlorpyrifos into other metabolites. One such metabolite resulting from hydrolysis is 3,5,6-tichloro-2-pyridinol (TCP) having a half life of 65–360 days (Briceno et al. 2012; Das and Adhta 2015; Singh et al. 2004). However, TCP is an antimicrobial metabolite which inhibits the proliferation of microorganisms in the soil and also prevents its own degradation by microorganisms and chlorpyrifos degradation (Racke 1993). Environmental protection agency (EPA) of the USA has listed TCP as a potential endocrine disrupting chemical (US EPA. 2009). Hence, there is a need of detoxify this pollutant from the environment. *Alcaligens faecalies* (Yang et al. 2005), *Enterobacter* sp. (Singh et al. 2003), *Pseudomonas nitroreducens* PS-2 (Korade and Fulekar 2009), *Serratia* sp. (Xu et al. 2007), *Sphingomonas* sp. (Li et al. 2007), and *Stenotrophomonas* sp. (Yang et al. 2006) all these are the papers which reports the diversity of microbes capable of complete mineralization of chlorpyrifos without the formation of TCP.

The current work was carried out to assess the effect of chlorpyrifos on soil microbial diversity and biotransformation of chloropyrifos using pure microbial isolate.

## Materials and methods

### Chemicals

Analytical grade Chlorpyrifos and 3,5,6-tichloro-2-pyridinol was obtained from Sigma-Aldrich Co., USA. Stock solution was prepared in HPLC grade Acetonitrile. All other Microbiological media used in this study were purchased from Hi-Media, Mumbai, India.

### Soil sampling

Soil samples were collected in polythene bags from the surface layer of 0–15 cm from Chamundi Hills, Located in Mysore, Karnataka. The soil has never been exposed to any insecticides in the past. The collected soil samples were air dried, sieved through a 2 mm mesh, and stored in 4 °C until further use.

### Chlorpyrifos treatment

The soil sample was placed in a Petri dish, and each dish was treated with CP to give a final concentration of 100 and 200 µg/g separately, and the contents were mixed gently and incubated at room temperature for 1, 7, and 14 days, respectively. The moisture content of soil was kept by adding sterile distilled water at regular intervals to obtain their original weight. All the experiments were carried out in triplicates along with a control plate which received only sterile water. The treated soil samples were analyzed to study the insecticide effect on microbial diversity by plate count technique. The observations were recorded and compared with control plates.

### Total counts

Total soil microflora was counted by a soil dilution plate technique, using nutrient agar (Hi-Media) for bacteria and fungal agar (Hi-Media) for fungi. The inoculated agar plates for bacteria were incubated for 24–48 h at 37 °C and 3–4 days at room temperature for fungi.

### Biodegradation of chlorpyrifos by soil bacteria

The strain designated as HP-11 showing luxuriant growth on fungal agar plate from CP treated soil was further selected for biodegradation studies through the enrichment method. MSM medium (in grams per liter) 1.5 g K_2_HPO_4_, 0.5 g KH_2_PO_4_, 0.2 g MgSO_4_·7 H_2_O, 0.5 g NaCl, and 1.5 g NH_4_NO_3_ was used for degradation test. Erlenmeyer flasks (250 ml) containing 100 ml MSM medium were supplemented with 100 mg/l of Chlorpyrifos as the sole carbon source and inoculated with HP-11 cell suspension after centrifugation at 10,000 RPM for 5 min by 10 ml of overnight culture grown in LB broth and incubated at 30 °C in a shaker for 14 days. The test was performed in triplicate, along with uninoculated flasks as a control.

### LC–MS analysis

After 14 days of incubation, 35 ml culture aliquot was taken in 50 ml centrifuge tube and centrifuged at 10,000 RPM for 5 min. Later, the supernatant was taken in separatory flask and extracted using equal volumes of n-Hexane by the shake flask method, and the organic aqueous layer was separated. The solvent was evaporated by rotary evaporator. The residue was then dissolved in HPLC grade acetonitrile and analyzed using liquid chromatography-mass spectroscopy (LC–MS) (Acquity Waters, USA). The LC–MS was equipped with a BEHC 181.7 µm column (10 × 50 mm) with auto injector. The cartridges were conditioned with acetonitrile and washed with deionized water containing 0.1 % formic acid. Mass spectroscopy (MS) was performed using a Synapt G2 HPMS MS (Waters, USA) equipped with Electron spray ionization (ESI) detector. The operating condition was Capillary (kV)-3.00, sampling cone-40.00, extraction cone-4.00, source temperature (°C)-100, desolvation temperature (°C)-200, and desolvation gas flow (l/h)-500.0.

### Identification and characterization

The strain showing *Actinomycete* appearance on a Petri dish containing fungal agar designated as HP-11 was streaked on Kenknight and Munaier’s media (pH 7.2) containing (g/l) 0.1 g KH_2_PO_4_, 0.1 g MgSO_4_, 0.1 g NaNO_3_, 0.1 g KCl, 10 g Dextrose, and 15 g Agar (Sasikala et al. 2012). The Morphology was investigated by light microscope (Labomed, LX, 400) and Scanning electron microscope (Zeiss EVO LS 15). Various biochemical tests of the strain were carried out and compared with Bergey’s manual of systematic bacteriology.

## Results

### Effect of chlorpyrifos on soil microorganisms

The soil sample collected from the forest area of Chamundi hill was found to be fertile by the study of its Physical–chemical characteristics. The soil had the neutral pH 7 with organic Carbon content higher than 0.75 %. The available nitrogen in the soil was 4–7 kg/acre, and the phosphate content of the soil was 11–15 kg/acre.

**Fig. 1:**
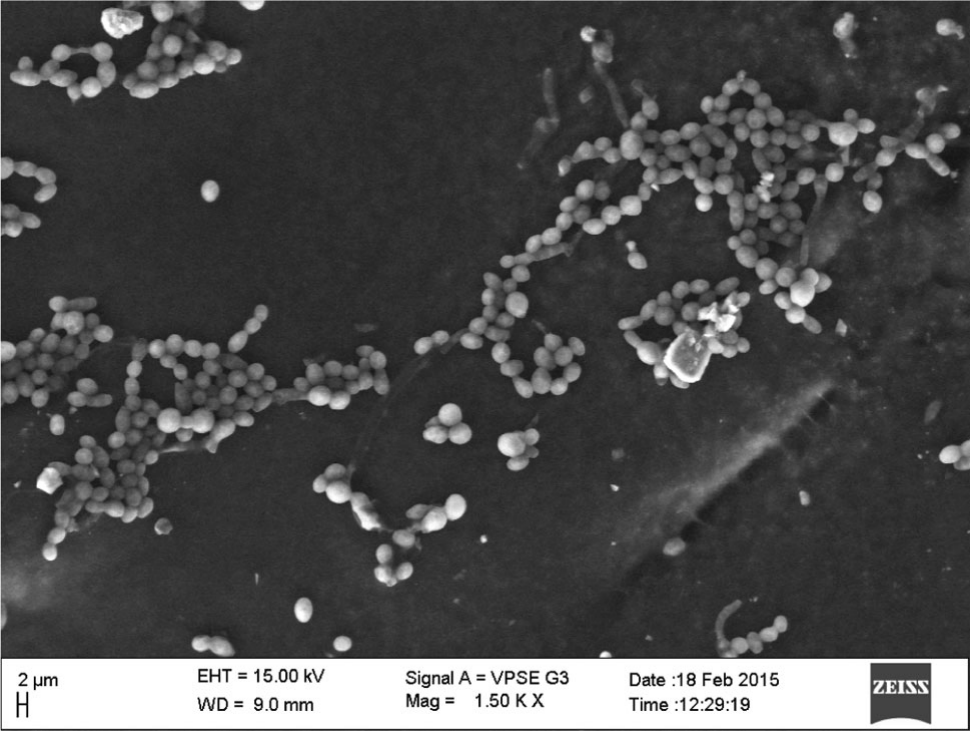
Scanning electron micrograph showing spore chain morphology of *Streptomyces* sp. HP-11

The effect of chlorpyrifos on soil microorganisms was analyzed at periodic intervals with soil samples treated with two different concentrations 100 and 200 µg/g of CP and compared with the control sample for the determination of insecticide effect. Observation on day one showed that insecticide inhibited number of colony forming units of both bacteria and fungi. Microbial colonies were highly varied at 200 µg/g concentration (Tables 1, 2). On the 7th day observation, improvement in microbial population was observed in soil samples of both concentrations, and number of bacterial colonies increased by five times when compared with the first day result. However, only few numbers of colonies increased in the case of fungi. On the 14th day, the bacterial population was almost recovered, and the numbers of colonies were found to be almost equal with the control soil. However, in the case of fungi, the fungal diversity was highly dominated by a single *Actinomycete* species which belonged to genus *Strepotomyces* by the study of its colony characteristics and microscopic observation and according to Kumar et al. (2011) and it is shown in Fig. 1. The colonies that grew on the Kenknight and Munaier’s media were slow growing, aerobic with white aerial mycelia. The species was Gram positive, spore forming, and pigment producer. A confirmatory identification to genus *Streptomyces* was based on biochemical test performed according to Taddei et al. (2006) (data not shown).

**Table 1:** Number of Colony forming units of bacteria observed on nutrient agar plates with soil treated with 100 and 200 µg/g chlorpyrifos and control

S. no	Concentration of pesticide ammended to the soil sample	Dilution	Day 1 CFU/ml	Day 7 CFU/ml	Day 14 CFU/ml
1	Control	10^−2^	142	T.N.C	312
		10^−3^	86	T.N.C	224
		10^−4^	16	T.N.C	173
2	100 µg/g	10^−2^	44	197	T.N.C*
		10^−3^	16	117	202
		10^−4^	4	41	158
3	200 µg/g	10^−2^	10	51	T.N.C
		10^−3^	0	30	172
		10^−4^	0	18	120

* Too numerous to count, values are mean of three experiments

**Table 2:** Number of Colony forming units of dominant *Actinomycete* colonies observed on fungal agar plates of soil treated with 100 and 200 µg/g chlorpyrifos and control

S.no	Concentration of pesticide ammended to the soil sample	Dilution	Day 1 CFU/ml	Day 7 CFU/ml	Day 14 CFU/ml
1	Control	10^−1^	74	57	65
		10^−2^	21	21	14
		10^−3^	12	6	4
2	100 µg/g	10^−1^	43	54	112
		10^−2^	16	21	54
		10^−3^	6	6	18
3	200 µg/g	10^−1^	29	10	142
		10^−2^	9	4	78
		10^−3^	2	1	30

Values are mean of three experiments

*Actinomycete*s are a group of Gram-positive bacteria exhibiting few fungal characters. The number of *Actinomycete* colonies found in pesticide applied soil sample is higher than the number of colonies found in control. The number of colonies found in concentration 200 µg/g was higher than 100 µg/g, and overall fungal diversity was inhibited and dominated by only one species of *Actinomycete*. However, in control soil sample, there was rich fungal diversity of *Cephalosporium, Penicellium, Aspergillus, Fusarium, Cladosporium*, and *Trichoderma* species. This may indicate that *Streptomyces* sp. HP-11 has the ability to utilize the chlorpyrifos as its carbon source.

### Biotransformation of chlorpyrifos

**Fig. 2:**
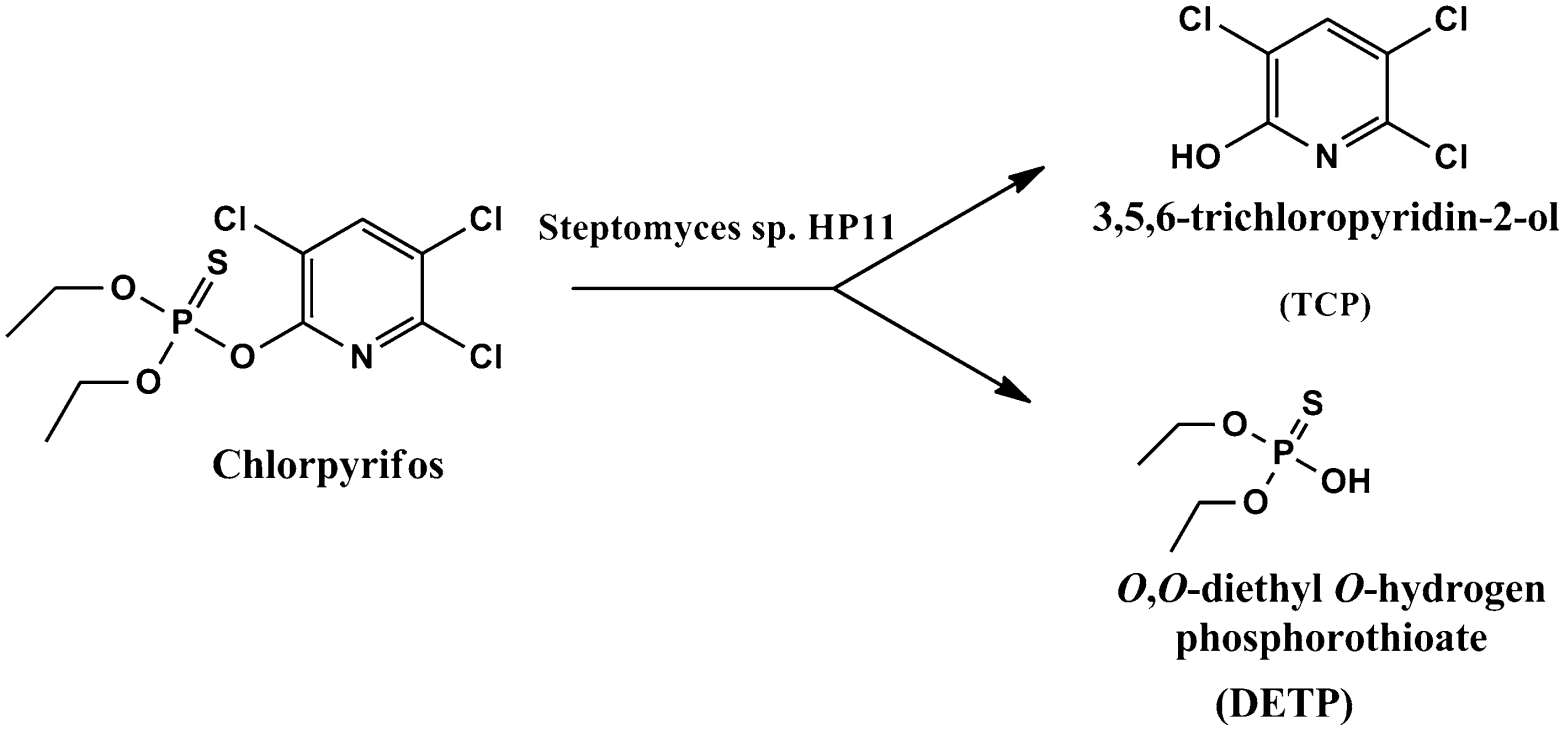
Proposed biodegradation pathway of *Streptomyces* sp. HP-11

**Fig. 3:**
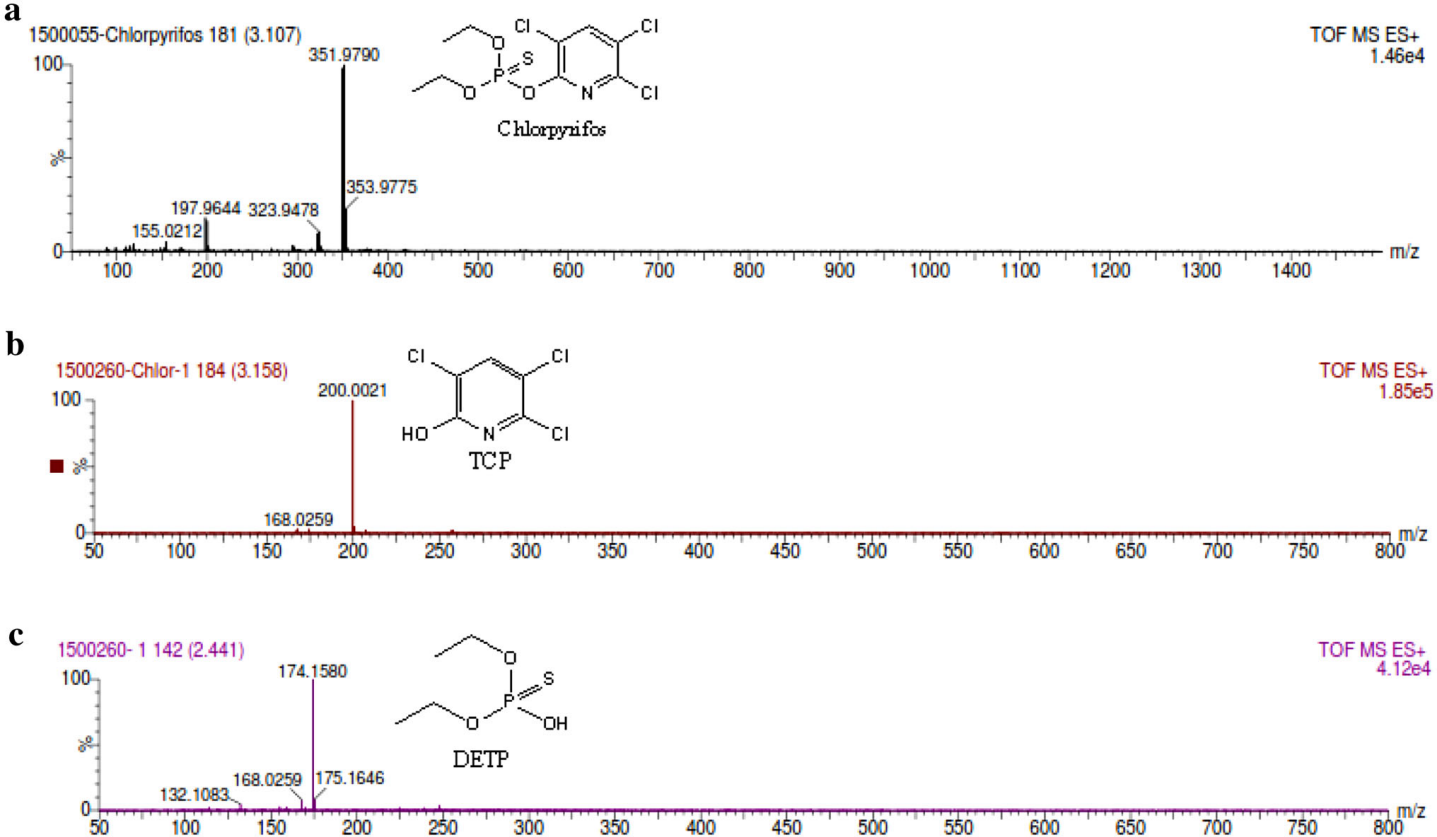
LC-MS spectrum of CP and biotransformed products. **a** Mass spectrum of the standard Chlorpyrifos (100 mg/l), **b** mass spectra of metabolite TCP, and **c** mass spectra of metabolite DETP

Biotransformation of chlorpyrifos into its metabolites was analyzed by Liquid Chromatography-Mass Spectroscope (LC–MS). Based on LC-MS analysis, degradation pathway of Chlorpyrifos by *Streptomyces* HP-11 has been proposed in Fig 2. The retention time was 3.10 min for standard with *m*/*z*+1 of 351.9, as shown in (Fig. 3a). Further LC–MS analysis of 14 days sample showed the formation of two new peaks with *m/z*+1 of 200 and 174 (Fig. 3b, c) which corresponds to mass value to 3,5,6-tichloro-2-pyridinol (TCP) and *O*,*O*-diethyl *O*-hydrogen phosphorothioate (DETP), respectively, which was later metabolized into unknown polar metabolites. The strain HP-11 could use CP as sole carbon source in MSM media and degraded parental compound by forming its metabolites TCP and DETP through hydrolysis. However, these two new peaks were transient and disappeared later. The formation of TCP by streptomyces sp. in soil was the main factor for inhibition of other species of fungi due its antimicrobial property.

## Discussion

The applied insecticide persists in the soil for long periods and has negative impacts on soil microbial flora, killing or inhibiting certain specific groups of microorganisms (Araujo et al. 2003). In the present work, the response of soil microorganisms to Chlorpyrifos treatment at 100 and 200 µg/g concentrations was variable in alteration in microbial diversity of dominant *Actinomycete* species. The large numbers of microorganisms inhibited were fungi, due to the formation of e TCP through hydrolysis by *Streptomyces* sp. HP-11. This is to our knowledge is the first report that describes the effect of chlorpyrifos on soil fungal diversity. The application of chlorpyrifos favored *Actinomycete* growth, thereby inhibiting other microorganisms. However, Martinez-Toledo et al. (1992) had shown that Chlorpyrifos at concentration 10–300 µg/g significantly decreased aerobic dinitrogen fixing bacteria and also dinitrogen fixation but no effect on fungi and denitrifying bacteria. This may be due to pesticides, application results in decrease in certain groups of soil microorganisms and also increase in the population of certain resistant and dominant microorganisms, Kalia and Gosal (2011).

Detoxification of chlorpyrifos in soil and aquatic environments using microorganisms is a viable option with its eco-friendliness, high efficiency and cost effectiveness (Dhanya 2014). For successful bioremediation of contaminated site, customized bioaugmenting agent is well required (Tyagi et al. 2011). However, there are only few reports, which show effective biodegradation of TCP. Lakshmi et al. (2008) reported biotransformation of chlorpyrifos (92 %) by *Pseudomonas aeroginosa*, where TCP was the major metabolite after 20 days of incubation and disappeared after 30 days by forming unknown polar metabolites. Similarly, fungal isolate *Cladosporium cladosporioids* Hu-01 isolated from activated sludge, biotransformed CP into TCP in 5 days, and later disappeared quickly (Chen et al. 2012). *Ralstonia* sp. metabolized 100 mg/l TCP within 12 h and 700 mg/l in 80 h by forming 3,6-dihydroxypyridine-2-dione as the green metabolite (Li et al. 2010). *Paracoccus* sp.TRP could utilize both CP and TCP as the sole carbon and nitrogen source resulting in complete mineralization (Xu et al. 2008). Our strain HP-11 which tolerated high concentration and also inhibited other groups of fungi was also able to biotransform CP into TCP and DETP after 14 days incubation in MSM media, which later disappeared into unknown polar metabolites. This result is similar to the previous findings (Abraham et al. 2013), where the Actinobacterial strain *Gordonia* sp.JAAS1 was able to degrade 110 mg/l of Chlorpyrifos within 24 h of incubation by forming TCP which finally degraded into DETP after 72 h, and *Bacillus Subtilis* Y242 isolated from agricultural waste water was able to degrade 95.2 % of 150 mg/l of Chlorpyrifos within 48 h, where 3,5,6-trichloro-2-methoxypyridine (TMP) was major transformed product (El-Helow et al. 2013).

## Conclusion

The applied insecticide persists in the soil for a long period and has negative impacts on soil microbial flora, resulting in the change of microbial diversity. In the present work, the effect of organophosphorous insecticide chlorpyrifos (CP) on soil microbial population was assessed by cultivable method. A number of microorganisms, such as fungi and bacteria, were inhibited by CP. The application of CP favored the *Actinomycete* growth in the soil, thereby inhibiting other microorganisms. Biotransformation studies showed that *Streptomyces* sp. HP-11 could degrade both CP and TCP, which can be used to clean up chlorpyrifos contaminated sites.

## Electronic supplementary material

Below is the link to the electronic supplementary material.

Supplementary material 1 (DOCX 476 kb)

## References

[CR1] Abraham J, Shanker A, Silambarasan S (2013). Role of *Gordonia* sp JASS1 in biodegradation of chlorpyrifos and its hydrolysing metabolite 3,5,6-Trichloro-2-pyridinol. Lett Appl Microbiol.

[CR2] Andreu V, Pico Y (2004). Determination of pesticides and their degradation products in soil: critical review and comparison of methods. Trends Anal Chem.

[CR3] Araujo ASF, Monteri RTR, Abarkeli RB (2003). Effect of glyphosate on the microbial activity of two Brazilian soils. Chemosphere.

[CR4] Briceno G, Fuentes MS, Palma G, Jorquera MA, Amoroso MJ, Diez MC (2012). Chlorpyrifos biodegradation and 3,5,6-trichloro-2-prridinol production by *Actinobacteria* isolated from soil Int. Biodeter Biodegr.

[CR5] Chen S, Liu C, Peng C, Liu H, Hu M, Zhong G (2012). Biodegradation of Chlorpyrifos and its Hydrolysis Product 3,5,6-Trichloro-2-Pyridinol by a New Fungal Strain *Cladosporium cladosporioides* Hu-01. PLoS ONE.

[CR6] Das S, Adhta TK (2015). Degradation of Chlorpyrifos in tropical rice soils. J Environ Manag.

[CR7] Dhanya MS (2014). Advances in microbial biodegradation of chlorpyrifos. J Environ Res Dev.

[CR8] El-Helow ER, Badawy MEI, Mabrouk MEM, Mohamed EAH, Beshlawy EYM (2013). Biodegradation of Chlorpyrifos by a newly isolated *Bacillus subtilis* strain, Y242. Bioremediation J.

[CR9] Kalia A, Gosal SK (2011). Effect of pesticide application on soil microorganisms. Arch Agron Soil Sci.

[CR10] Korade DL, Fulekar MH (2009). Rhizosphere remediation of chlorpyrifos in mycorrhizospheric soil using ryegrass. J Hazard Mater.

[CR11] Kumar V, Bharti A, Gusain O, Bisht GS (2011). Scanning electron microscopy of *Streptomyces* without use of any chemical fixatives. Scanning.

[CR12] Lakshmi CV, Kumar M, Khanna S (2008). Biotransformation of chlorpyrifos and bioremediation of contaminated soil. Int Biodeter Biodegr.

[CR13] Li X, He J, Li S (2007). Isolation of a chlorpyrifos-degrading bacterium, *Sphingomonas* sp. strain Dsp-2, and cloning of the mpd gene. Res Microbiol.

[CR14] Li X, Jiang J, Gu L, Ali SW, He J, Li S (2008). Diversity of chlorpyrifos-degrading bacteria isolated from chlorpyrifos-contaminated samples. Int Biodeter Biodegr.

[CR15] Li J, Liu J, Shen W, Zhao X, Hou Y, Cao H, Cui Z (2010). Isolation and characterization of 3,5,6-trichloro-2-pyridinol degrading Ralstonia sp. strain T6. Bioresour Technol.

[CR16] Lo CC (2010). Effect of pesticides on soil microbial community. J Environ Sci Health, Part B.

[CR17] Martinez-Toledo MV, Salmeron V, Gonzalez-Lopez J (1992). Effect of insecticides methylpyrimifos and chlorpyrifos on soil microflora in an agricultural loam. Plant Soil.

[CR19] Racke KD (1993). Environmental fate of chlorpyrifos: review. Environ Contam Toxicol.

[CR20] Sasikala C, Jiwal S, Rout P, Ramya MC (2012). Biodegradation of chlorpyrifos by bacterial consortium isolated from agricultural soil. World J Microbial Biotechnol.

[CR21] Singh BK, Walker A (2006). Microbial degradation of organophosphorous compounds. FEMS Microbiol Rev.

[CR22] Singh BK, Walker A, Morgan JA, Wright DJ (2003). Effects of soil pH on the biodegradation of chlorpyrifos and isolation of a chlorpyrifos-degrading bacterium. Appl Environ Microbiol.

[CR23] Singh BK, Walker A, Morgan JAW, Wright DJ (2004). Biodegradation of Chlorpyrifos by *Enterobacter* Strain B-14 and its use in Bioremediation of Contaminated Soils. Appl Environ Microbiol.

[CR24] Taddei A, Rodriguez MJ, Vilchez EM, Castelli C (2006). Isolation and identification of *Streptomyces* spp. from venezuelan soils: morphological and biochemical studies. Microbial Res.

[CR25] Tyagi M, Manuela MR, Fonseca D, Carla C, Carvalho D (2011). Bioaugmentation and biostimulation strategies to improve the effectiveness of bioremediation processes. Biodegradation.

[CR26] US EPA (2009) Endocrine disruptor screening program (EDSP). US EPA, Washington,DC

[CR27] Xu GM, Li YY, Zheng W, Peng X, Li W, Yan YC (2007). Mineralization of chlorpyrifos by co-culture of *Serratia* and *Trichosporon* sp. Biotechnol Lett.

[CR28] Xu G, Zheng W, Li Y, Wang S, Zhang J, Yan Y (2008). Biodegradation of chlorpyrifos and 3,5,6 trichloro-2-pyridinol by a newly isolated *Paracoccus* sp. strain TRP. Int Biodeter Biodegr.

[CR29] Yang L, Zhao Y, Zhang B, Yang C, Zhang X (2005). Isolation and characterization of a chlorpyrifos and 3,5,6-trichloro-2-pyridinol degrading bacterium. FEMS Microbiol Lett.

[CR30] Yang C, Liu N, Guo XM, Qiao CL (2006). Cloning of mpd gene from a chlorpyrifos degrading bacterium and use of this strain in bioremediation of contaminated. FEMS Microbiol Lett.

