# Supplementary material for: Effect of chlorpyrifos on soil microbial diversity and its biotransformation by Streptomyces sp. HP-11
Source: 3 Biotech. 2016 Jun 24;6(2):147. doi: 10.1007/s13205-016-0462-2 (PMC4920701; doi:10.1007/s13205-016-0462-2)
Supplement: Supplementary file 1 — Supplementary material 1 (DOCX 476 kb) [file 13205_2016_462_MOESM1_ESM.docx]

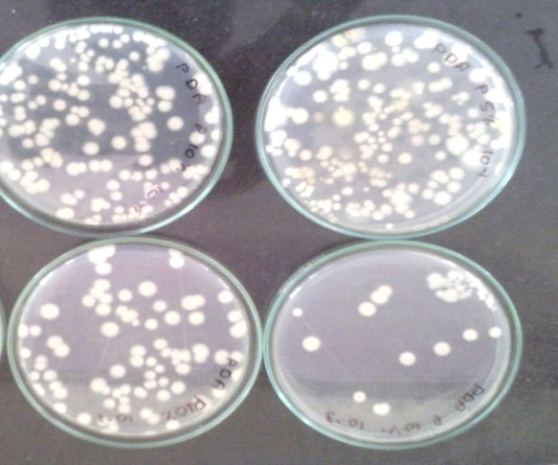


Fig 1 *Actinomycetes* colonies growth on Fungal Agar plates of soil sample treated with 200µg/l of Chlorpyrifos after 14 days


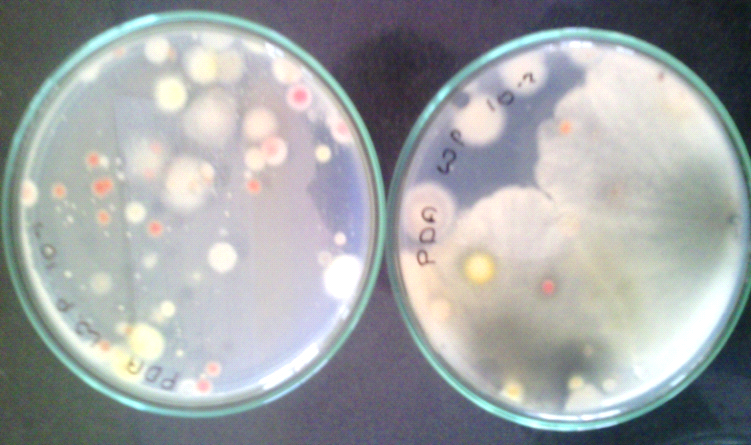


Fig 2 different fungal colonies found on fungal agar plates of Control soil sample
